# Metagenome-Assembled Genomes Isolated from a Human Fecal Diarrhea Sample

**DOI:** 10.1128/MRA.00234-21

**Published:** 2021-05-13

**Authors:** Tshepiso Pleasure Ateba, Kazeem Adekunle Alayande, Ngoma Lubanza, Mulunda Mwanza

**Affiliations:** a Department of Animal Health, Faculty of Natural and Agricultural Sciences, Mafikeng Campus, North West University, Mmabatho, South Africa; b Department of Microbiology, Faculty of Natural and Agricultural Sciences, Mafikeng Campus, North West University, Mmabatho, South Africa; University of Maryland School of Medicine

## Abstract

Diarrheal infection is the second leading infectious disease that is killing children under the age of 5 years. This study investigates the microbial community within a fecal sample from a diarrhea-affected child through shotgun metagenomic sequencing.

## ANNOUNCEMENT

Diarrheal infection is a significant public health crisis and a common infection among children under the age of 5 years, with a high mortality rate. It is the second leading killer of children, largely due to dehydration and associated compromised immunity and malnutrition (1). A stool sample from a diarrhea-affected child was collected at Mafikeng Provincial Hospital, South Africa (25.88S, 25.67E), and metagenome DNA was extracted from roughly 150 mg of the sample using the Quick-DNA fecal/soil microbe miniprep kit (Zymo Research, USA). A library was prepared with the Nextera DNA Flex kit using Nextera DNA CD index adapters (96 indexes plated) and then was sequenced on an Illumina NovaSeq system. The number of reads generated was 16,261,498, with a read length of 2 × 150 bp and a sequencing depth of 31×.

The sequenced data generated were assessed and filtered for low-quality reads and adapter fragments using Trimmomatic v0.36 (2). The adapter sequences were clipped using a mismatch value of 2, a palindrome clip threshold of 30, and a simple clip threshold of 10. Taxonomic distribution of the metagenome was determined using Kaiju v1.7.2 (3) and GOTTCHA2 v2.1.6, and *de novo* metagenome assembly was performed with metaSPAdes v3.13.0 (4). The *de novo* assembly was binned using MaxBin 2 v2.2.4 (5) and CONCOCT v1.1 set at different modes (Bowtie2-default, Bowtie2-verysensitive, and BBMap), and results were optimized to exclude bins with low completeness and high contamination levels using DAS Tool v1.2. Each bin was then extracted as a metagenome-assembled genome (MAG), and quality was assessed using CheckM v1.0.18 (6). The taxonomic assignments were obtained for the MAGs based on the Genome Taxonomy Database using GTDB-Tk v1.1.0 (7).

The MAGs were annotated using the NCBI Prokaryotic Genome Annotation Pipeline (PGAP) v5.0 (8), and acquired drug resistance genes were determined using ResFinder v4.0 (9). Default parameters were used for all software engaged in the analysis except where stated otherwise.

The taxonomic distribution of the bacteria within the fecal metagenome indicates five different phyla, with the phylum *Firmicutes* having the largest proportion. Other identified phyla include *Proteobacteria*, *Bacteriodetes*, *Actinobacteria*, and *Chlamydiae*. The assembly size of the metagenome is 68,552,285 bp, contained in 9,659 contigs that were binned into 12 MAGs; these included pathogens such as Shigella flexneri, Prevotella stercorea, Peptostreptococcus stomatis, *Senegalimassilia* sp., and *Dialister* sp., which were previously isolated from human clinical samples (Table 1). Not all of the contigs were binned into the MAGs.

**TABLE 1:** Characteristics and accession numbers of the MAGs

MAG identity	Genome size (bp)^*a*^	Total no. of CDSs^*b*^	No. of pseudogenes	No. of contigs	*N*_50_ (bp)	G+C content (%)	Completeness (%)^*c*^	Contamination (%)^*c*^	GenBank accession no.
Shigella flexneri 00NH	4,591,307	4,403	182	108	60,177	50.65	99.50	0.39	JAEUGE000000000
Faecalibacterium prausnitzii 01NH	2,280,455	2,076	34	165	16,934	59.83	93.54	0.0	JAEUGF000000000
Peptostreptococcus stomatis 03NH	1,931,518	1,727	22	47	66,549	36.63	98.60	0.0	JAEUGG000000000
*Porphyromonas* sp. strain 10NH	2,101,215	1,647	42	178	13,454	52.76	96.46	0.31	JAEUGH000000000
*Dialister* sp. strain 12NH	2,082,611	1,772	32	254	9,599	52.76	96.20	2.61	JAEUGI000000000
*Mogibacterium* sp. strain 18NH	2,030,227	1,785	16	36	67,172	50.94	97.16	0.0	JAEUGJ000000000
*Blautia* sp. strain 23NH	3,409,943	3,163	65	408	10,913	41.33	86.95	2.53	JAEUGK000000000
*Senegalimassilia* sp. strain 28NH	2,246,693	2,035	33	294	7,956	62.26	87.90	0.61	JAEUGL000000000
Prevotella stercorea 30NH	2,629,523	2,182	21	99	39,258	49.63	94.76	0.96	JAEUGM000000000
*Holdemanella* sp. strain 41NH	1,765,938	1,901	46	316	5,463	33.90	70.75	1.65	JAEUGN000000000
Muribaculaceae bacterium 42NH	2,474,455	2,049	34	104	28,183	50.00	96.98	0.21	JAEUGO000000000
*Prevotellamassilia* sp. strain 51NH	1,756,596	1,420	38	357	4,830	50.70	67.49	0.37	JAEUGP000000000

a The genome sizes were determined using v1-KBase Genome Annotations. Assembly-5.0 and NCBI PGAP v5.0. CDS, coding sequence.

b CDSs, coding sequences.

c The values for completeness and contamination of each MAG were determined using CheckM v1.0.18.

Ethical clearance for the study was provided by the Research Ethics Committee of the North West University, South Africa (protocol NWU-00160-14-A9).

### Data availability.

All data were deposited under the GenBank BioProject number PRJNA684904. The whole-genome shotgun projects were deposited in DDBJ/ENA/GenBank under the accession number JAERMT000000000. The version described in this paper is the first version, JAERMT010000000. The SRA accession number for the raw reads is SRX9681389.
